# A novel vibriophage exhibits inhibitory activity against host protein synthesis machinery

**DOI:** 10.1038/s41598-020-59396-3

**Published:** 2020-02-11

**Authors:** Khrongkhwan Thammatinna, MacKennon E. Egan, Htut Htut Htoo, Kanika Khanna, Joseph Sugie, Jason F. Nideffer, Elizabeth Villa, Anchalee Tassanakajon, Joe Pogliano, Poochit Nonejuie, Vorrapon Chaikeeratisak

**Affiliations:** 1 Center of Excellence for Molecular Biology and Genomics of Shrimp, Department of Biochemistry, Faculty of Science, Chulalongkorn University, Bangkok, 10330 Thailand; 2 Division of Biological Sciences, University of California, San Diego, La Jolla, California, USA; 3 Institute of Molecular Biosciences, Mahidol University, Salaya, Nakhon Pathom Thailand

**Keywords:** Bacteriophages, Phage biology

## Abstract

Since the emergence of deadly pathogens and multidrug-resistant bacteria at an alarmingly increased rate, bacteriophages have been developed as a controlling bioagent to prevent the spread of pathogenic bacteria. One of these pathogens, disease-causing *Vibrio parahaemolyticus* (VP_AHPND_) which induces acute hepatopancreatic necrosis, is considered one of the deadliest shrimp pathogens, and has recently become resistant to various classes of antibiotics. Here, we discovered a novel vibriophage that specifically targets the vibrio host, VP_AHPND_. The vibriophage, designated Seahorse, was classified in the family *Siphoviridae* because of its icosahedral capsid surrounded by head fibers and a non-contractile long tail. Phage Seahorse was able to infect the host in a broad range of pH and temperatures, and it had a relatively short latent period (nearly 30 minutes) in which it produced progeny at 72 particles per cell at the end of its lytic cycle. Upon phage infection, the host nucleoid condensed and became toroidal, similar to the bacterial DNA morphology seen during tetracycline treatment, suggesting that phage Seahorse hijacked host biosynthesis pathways through protein translation. As phage Seahorse genome encodes 48 open reading frames with many hypothetical proteins, this genome could be a potential untapped resource for the discovery of phage-derived therapeutic proteins.

## Introduction

*Vibrio* is a genus of motile Gram-negative bacteria that possesses a curved-rod cell shape with a single flagellum. Vibrios are abundant and diverse bacteria that are typically found in marine habitats. The genus *Vibrio* consists of 14 recognized clades and at least 86 different species^1^. While some of them are not pathogenic, many can cause serious health effects in both human and aquatic life. Due to the continuously rising ocean temperature, the composition of vibrio in the ocean microbiome has been reported to be higher than usual^2–4^. This vibrio-rich environment might increase the incident of a vibrio outbreak in the near future posing risks to global health^2^.

*Vibrio parahaemolyticus*, which is one of the disease-causing *Vibrio* species, is pathogenic to both humans and marine animals^5^. Consumption of raw seafoods contaminated with the bacteria can cause acute gastroenteritis^5,6^. This opportunistic bacterium is also able to infect through an open wound which can lead to sepsis and, in rare cases, subsequent death in immunocompromised patients^7,8^. Moreover, *V. parahaemolyticus* that has acquired a plasmid encoding the deadly binary toxins PirA^vp^/PirB^vp^ is even more virulent^9^. The *V. parahaemolyticus* strain harboring the plasmid has been found to cause a newly emerging disease in shrimp, known as acute hepatopancreatic necrosis disease (AHPND)^9^. Moreover, the AHPND-causing plasmid is also found to be transferable among other vibrios, increasing the chance of the disease spreading regionally and globally^10^. Unsurprisingly, the spread of AHPND has been reported in many countries, including China, Vietnam, Malaysia, Thailand, Mexico, the Philippines, and South America^11–13^. Because of its efficient transferability, the gross impact of the infection is also a concern. The infection from AHPND-causing *V. parahaemolyticus* (VP_AHPND_) in cultured shrimp results in a near 100% mortality rate within a week after the first symptoms appear^14^. Altogether, VP_AHPND_ has easily become a leading cause in tremendous reduction of shrimp farming yield, which could lend itself to global financial detriments in key shrimp aquaculture industries.

To prevent these detriments, antibiotics are top candidate for control agents because of their ease of use and high accessibility in many countries. However, the heavy misuse of antibiotics undoubtedly contributes to the emergence of multidrug-resistant (MDR) bacteria. This also accelerates the spread of multidrug resistant genes to other bacteria via well documented genetic element transfers within the microbial community^15^. As a result, over the last decade, MDR-vibrios (MDR-V) have been rapidly emerging worldwide and have been found in the United States, China, India and South East Asian countries including Thailand^16,17^, with the most recent emergence in Nigeria and Malaysia this past year^18–20^. In particular, MDR-V isolated from cultured animals are now strongly resistant to ampicillin and tetracycline, and moderately resistant to nalidixic acid^16,17^. Specifically, VP_AHPND_ strains have been reported to be resistant to ampicillin, tetracycline, and erythromycin^21,22^. Thus, alternative measures are desperately needed in order to control vibrio outbreaks and prevent further antibiotic resistance development.

Bacteriophage therapy has been under a recent spotlight as an alternative therapeutic method that helps minimize the extensive use of antibiotics, delay the emergence of antibiotic resistance, and combat the existing MDR bacteria^23^. In aquaculture, phage application using various potent lytic phages has been proven successful in preventing vibriosis by *V. harveyi*, *V. alginolyticus*, *V. coralliilyticus*, *V. anguillarum*, *V. cyclitrophicus*, *V. splendidus* as well as *V. parahaemolyticus*^24,25^. During the past few years, many phages, such as VP882, Vp1, Vpms1, A3S, VpKK5, and VVP1, have been isolated from natural resources exhibiting potent antibacterial activity against *V. parahaemolyticus*^26^. However, due to the high specificity of phage, this renders their use too narrow and thus impractical. There have been recent studies to address this, one of which utilized a phage cocktail comprising three lytic phages simultaneously (VP-1, VP-2, and VP-3) that inactivated *V. parahaemolyticus* more efficiently than the individual phages alone^27^. Additionally, a series of reports from Jun JW *et al*. revealed that pVp-1 showed a bactericidal activity toward MDR-*V. parahaemolyticus* and a broad-host range against VP_AHPND_ strains obtained from diverse regions. Moreover, its application on VP_AHPND_-infected penaeid shrimp in a lab-scale tank revealed high effectiveness in both prophylactic and therapeutic aspects indicating the potential method of phage therapy, so there is evidence that phage therapy can overcome the specificity concern^28–30^.

With the ultimate goal of extending the variety of phages targeting VP_AHPND_ and to provide more untapped resources for antimicrobial discovery from phage-encoded products, we report here a novel vibriophage isolated from seawater that was able to kill VP_AHPND_ strain TM. Exploiting fluorescence microscopy techniques based on bacterial cytological profiling (BCP) principle^31^, we observed a mechanism of pre-killing (MOK) of this vibriophage in which the phage intercepts host protein translation machinery during the period of infection before host cell lysis. Our study suggests the discovery of a possible therapeutic agent derived from the phage that inhibits protein synthesis of this pathogenic bacterium.

## Results

### Morphological and biological properties of phage Seahorse

**Figure 1:**
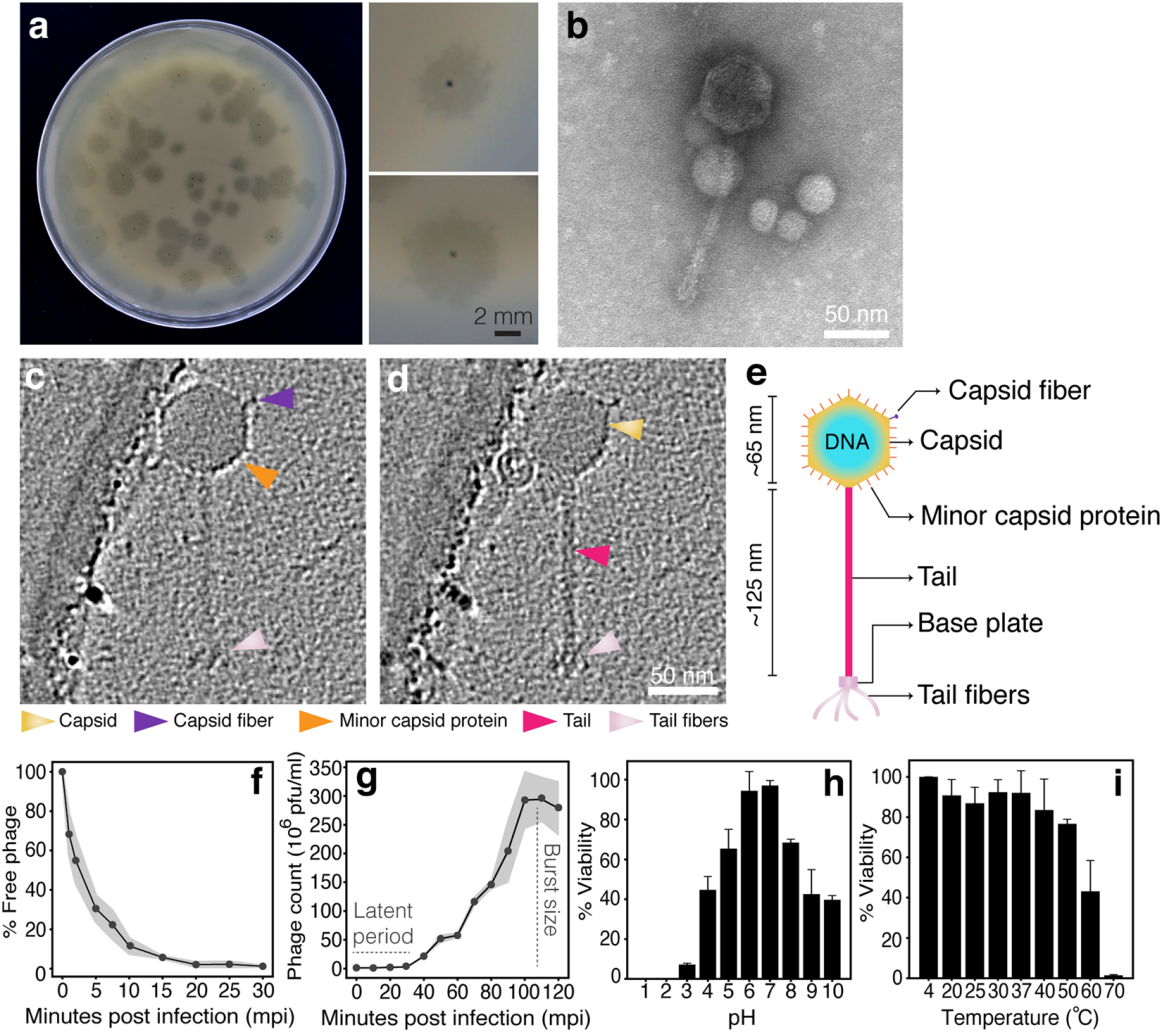
Morphological and biological properties of phage Seahorse. (**a**) Plaque morphology of phage Seahorse. An individual plaque is shown in the right panels. Scale bar equals to 2 mm. Morphology of phage Seahorse as determined by Negative staining and TEM (**b**) and Cryo-ET (**c**,**d**). Different slices (**c**,**d**) through the same Cryo-ET showing the structure of phage Seahorse. Arrows indicate capsid, capsid fiber, minor capsid protein, tail and tail fibers. Scale bar equals to 50 nm. (**e**) Schematic of phage Seahorse as visualized by Cryo-ET with the capsid size and the tail length indicated. (**f**–**i**) Biological studies of phage Seahorse; Adsorption assay (**f**), One-step growth curve (**g**), phage viability in different pH (**h**) and different temperature (**i**). The experiments (**f**–**i**) were conducted in at least 3 independent biological replicates and the data are represented as the mean ± standard deviation.

Bacteriophages that target VP_AHPND_ were enriched and isolated from seawater collected from a local shrimp farming area in Thailand. A phage selected for this study actively lysed VP_AHPND_ and produced a 2–3 mm plaque with a 0.5 mm-clear spot at the center surrounding by the halo-turbid area (Fig. 1a). As observed in negative staining by transmission electron microscopy (TEM), the phage belongs to the order *Caudovirales* and the family *Siphoviridae* as it has an icosahedral capsid with a long non-contractile tail with short tail fibers (Fig. 1b). In order to visualize the phage at a higher resolution and at a near-native state, we imaged the phage using cryo-electron tomography (cryo-ET). Our cryo-ET images indicated that the phage had a capsid of ~65 nm in diameter and a tail of ~125 nm in length (Fig. 1c–e; n = 3). The phage capsid seemed to be decorated with other proteins which may likely constitute the head fiber similar to those found in *Bacillus subtilis* phage Phi29 and adenoviruses^32–34^. Our cryo-ET images of the phage also revealed the presence of capsid fibers (the shaft with a knob) of ~10 nm in length at the vertices of the capsid (Purple arrows; Fig. 1c). We also see densities on the capsid facets (~3 nm) which may correspond to minor capsid proteins similar to those found in adenoviruses (Orange arrows; Fig. 1c)^34,35^. These are believed to enhance capsid stability by forming strong protein-protein interactions. Further biochemical and structural studies are needed to characterize and resolve these protein densities unambiguously. Based on the 3-dimensional phage structure under Cryo-ET (Movie S1), we designated this vibriophage as “Seahorse”.

To gain more information on the biological properties of phage Seahorse, we tested its host range and conducted a one-step growth curve, a phage tolerance test and measured phage adsorption rate. Out of 26 different bacterial strains tested, phage Seahorse exhibited a narrow host spectrum and specifically infected *V. parahaemolyticus* strain TM that causes AHPND or VP_AHPND_ (Table 1). A phage adsorption assay revealed that more than 95% of the phage were rapidly adsorbed onto the host cell within 15 minutes (Fig. 1f). The one-step growth curve showed that the phage propagated in the cell during the latent period for at least 30 minutes and resulted in a burst size of 72 virions per cell (Figs. 1g, S1, and Table S2). Additionally, the phage was highly tolerant to a wide range of pH and temperatures (Fig. 1h,i). Figure 1h revealed that the phage was able to infect the host with the highest infectivity at pH 6–7 and the infectivity was found to be completely lost at pH 1–3 (Fig. 1h). A thermal stability study showed that the phage was still active at temperatures between 4 °C–50 °C while the phage pre-treated with high temperatures above 60 °C significantly lost their infectivity (Fig. 1i).

**Table 1:** Host range determination of phage Seahorse.

Bacterial species	Strain	Source	Plaque formation
*Vibrio parahaemolyticus*	AHPND (TM)	Junprung *et al*.^73^	+
	Non-AHPND		−
	ATCC 17802	American type culture collection	−
	DMST 5665	DMST laboratory collection	−
*Vibrio harveyi*	Isolate 639	Department of Microbiology, Faculty of Science, Chulalongkorn University	−
	Isolate 102		−
	Isolate 2207		−
	Isolate 1526		−
	Isolate gn		−
	Isolate 35		−
*Vibrio alginolyticus*	DMST 14800	DMST laboratory collection	−
*Vibrio cholerae*	DMST 2873		−
*Vibrio fluvialis*	DMST 21248		−
*Vibrio vulnificus*	DMST 21245		−
*Vibrio mimicus*	DMST 21244		−
*Vibrio natrigens*	ATCC 14048	American type culture collection	−
Vibrio spp.*	VC1060	Isolated from healthy shrimp (This study)	−
	VC1061		−
	VC1062		−
	VC1063		−
*Pseudomonas aeruginosa*	PA01	Klockgether *et al*.^75^	−
*Pseudomonas chlororaphis*	200-B	Serwer *et al*.^76^	−
*Escherichia coli*	ATCC 25922	American type culture collection	−
*Burkholderia thailandensis*	ATCC 700388		−
*Acinetobacter baumannii*	ATCC 17978		−
	ATCC 196096		−

Different bacterial species and strains were used as the host to determine the host spectrum of the phage using a spot test.

*Identified by 16 s sequencing.

DMST: Department of Medical Sciences, Ministry of Public Health, Thailand.

### Genome features and annotation of phage Seahorse

**Figure 2:**
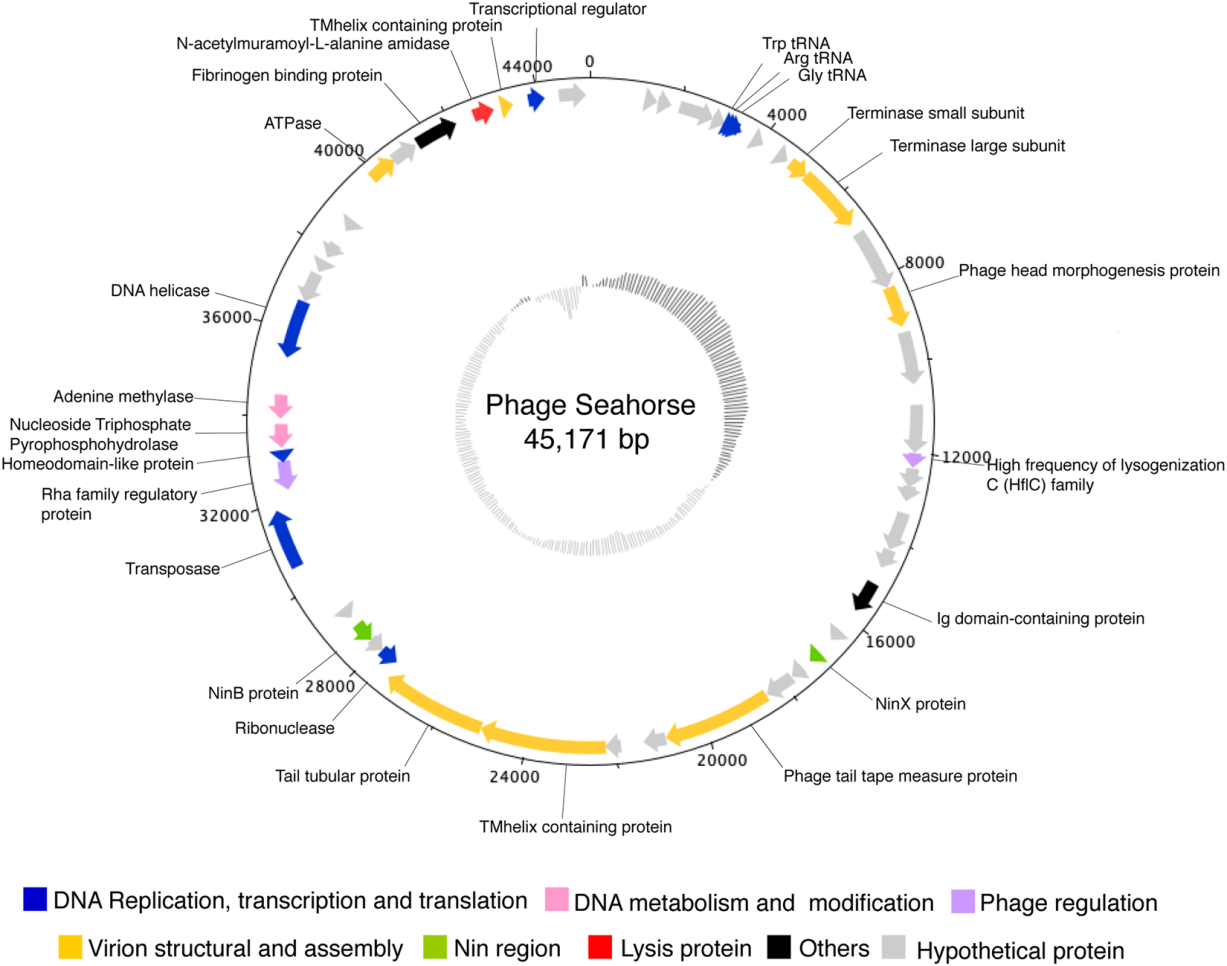
Genome map of phage Seahorse. The genome size is 45,171 base pairs long and the positions in term of base pairs are indicated by the number on the outermost circle. The grey scale on the innermost circle indicates GC content. The open reading frames (ORFs) are annotated and color-coded by their putative function; blue: DNA replication and transcription, and translation, pink: DNA metabolism and modification, purple: phage regulation, yellow: virion structural and assembly, green: Nin region, red: lysis protein, black: others, and grey: hypothetical proteins. The direction of arrows indicates gene arrangement in the genome.

The complete genome of Seahorse was 45,171 bp long with a GC content of 42.59% encoding 48 putative open reading frames (ORFs) and 3 tRNA genes (Fig. 2 and Table 2). These predicted ORFs were scattered throughout the phage Seahorse genome with different gene arrangements; 35 ORFs in the forward direction and 13 ORFs in the reverse direction (Fig. 2). Out of the total predicted ORFs, 22 ORFs were assigned a putative function according to the significant hits in the indicated databases with E-values less than 10^−4^ while the rest were identified as hypothetical proteins (Table 2). Among them, more than 80% of start codons in the ORFs were ATG followed by TTG (14.58%) and CTG (2.08%). We classified the 22 ORFs into 7 main groups according to function; (1) replication, transcription and translation, (2) DNA metabolism and modification, (3) virion structure and assembly, (4) phage regulation, (5) Nin regions, (6) lysis protein, and (7) other phage-related proteins (Fig. 2). In addition, we did not observe any antimicrobial resistance-coding genes or putative toxins from the phage genome.

**Table 2:** List of annotated proteins from ORFs in the genome of phage Seahorse.

ORF	Predicted function	Direction	Start	Stop	Size (n)	Sequence similarity	Accession no.	Database	E-value
ORF1	Hypothetical protein	+	1293	1496	204	Hypothetical protein NVP1103O_85 [Vibrio phage 1.103.O._10N.261.52.F2]	AUR87742.1	NCBI	6.00E-27
ORF2	Hypothetical protein	+	1514	1819	306	Hypothetical protein NVP1291O_36 [Vibrio phage 1.291.O._10N.286.55.F6]	AUS01750.1	NCBI	2.00E-46
ORF3	Hypothetical protein	+	2007	2786	780	Hypothetical protein NVP1263A_15 [Vibrio phage 1.263.A._10N.286.51.B1]	AUR99175.1	NCBI	3.00E-12
ORF4	Hypothetical protein	+	2787	3071	285	Hypothetical protein	168987	ACLAME	2.00E-07
ORF5	Hypothetical protein	+	3799	4032	234	Hypothetical protein [Vibrio phage LP.1]	AZU97916.1	NCBI	3.00E-14
ORF6	Hypothetical protein	+	4400	4681	282	Hypothetical protein ValSw33_31 [Vibrio phage ValSw3-3]	AVR75855.1	NCBI	3.00E-42
ORF7	Terminase small subunit	+	4761	5204	443	Phage(gi712916139); PHAGE_Shewan_3/49_NC_025466: terminase small subunit	PP_00071	PHASTER	7.35E-37
ORF8	Terminase large subunit	+	5194	6678	1484	phage(gi712915541); PHAGE_Shewan_1/41_NC_025458: terminase large subunit	PP_00070	PHASTER	0
ORF9	Hypothetical protein	+	6898	8274	1377	Hypothetical protein	184967	ACLAME	1.00E-71
ORF10	Phage head morphogenesis protein	+	8261	9190	929	MULTISPECIES: phage head morphogenesis protein [Vibrio]	WP_086959696.1	NCBI	1.00E-149
ORF11	Hypothetical protein	+	9312	10490	1179	Hypothetical protein	184946	ACLAME	2.00E-35
ORF12	Hypothetical protein	+	10936	12006	1071	Hypothetical protein	184931	ACLAME	3.00E-19
ORF13	High frequency of lysogenization C (HflC) family	+	12006	12332	327	High frequency of lysogenization C (HflC) family	YP_009275512.1	NCBI	1.33e-06
ORF14	Hypothetical protein	+	12339	12776	438	Hypothetical protein	184935	ACLAME	4.00E-13
ORF15	Hypothetical protein	+	12736	13089	354	Hypothetical protein	184932	ACLAME	2.00E-05
ORF16	Hypothetical protein	+	13355	14212	858	Hypothetical protein VPKG_00027 [Vibrio phage pYD21-A]	YP_007673989.1	NCBI	3.00E-71
ORF17	Hypothetical protein	+	14209	14652	444	Hypothetical protein ValSw33_31 [Vibrio phage ValSw3-3]	AVR75855.1	NCBI	3.00E-42
ORF18	Ig domain-containing protein	+	15028	15750	722	Uncharacterized conserved protein YjdB, contains Ig-like domain	COG5437	NCBI Conserved Domain Search	2.02E-05
ORF19	Hypothetical protein	+	16335	16598	264	Hypothetical protein NVP1116O_41 [Vibrio phage 1.116.O._10N.222.52.C10]	AUR88658.1	NCBI	2.00E-05
ORF20	NinX protein	+	16974	17261	287	NinX [Salmonella phage S102]	AXC39656.1	NCBI	7.00E-15
ORF21	Hypothetical protein	+	17466	17777	312	Hypothetical protein NVP1239O_45 [Vibrio phage 1.239.O._10N.261.52.F6]	AUR97481.1	NCBI	3.00E-08
ORF22	Hypothetical protein	+	17805	18461	657	Hypothetical protein	184973	ACLAME	3.00E-20
ORF23	Phage tail tape measure protein	+	18471	20900	2430	Lambda family phage tail tape measure protein	181776	ACLAME	5.00E-06
ORF24	Hypothetical protein	+	20900	21397	498	Hypothetical protein [Vibrio phage LP.2]	AZU97857.1	NCBI	3.00E-17
ORF25	Hypothetical protein	+	21903	22250	348	Hypothetical protein NVP1189B_19 [Vibrio phage 1.189.B._10N.286.51.B5]	AUR93845.1	NCBI	8.00E-23
ORF26	TMhelix containing protein	+	22238	25039	2801	TMhelix containing protein [Vibrio phage 1.110.O._10N.261.52.C1]	AUR88148.1	NCBI	3.00E-147
ORF27	Tail tubular protein	+	25039	27384	2345	tail tubular protein [Vibrio phage Athena1]	AUG84865.1	NCBI	2.00E-19
ORF28	Ribonuclease	−	27438	27878	441	Ribonuclease [Vibrio phage VaK]	ARH11752.1	NCBI	6.00E-46
ORF29	Hypothetical protein	−	27875	28228	354	Hypothetical protein NVP1254O_20 [Vibrio phage 1.254.O._10N.286.45.C8]	AUR98603. 1	NCBI	2.00E-20
ORF30	NinB protein	−	28225	28713	488	[Superfamily] cl21658 (PSSMID 328842) NinB protein	PRK09741	NCBI Conserved Domain Search	1.61E-37
ORF31	Hypothetical protein	−	28915	29139	225	Hypothetical protein ValSw33_20 [Vibrio phage ValSw3-3]	AVR75844.1	NCBI	5.00E-38
ORF32	Transposase	+	30555	31868	1313	PHAGE_Burkho_Bcep22_NC_005262: ISL3 family transposase; PP_00032; phage(gi38640338)	PP_00032	PHASTER	8.62E-64
ORF33	Rha family regulatory protein	−	32262	32933	671	Rha family regulatory protein [Vibrio phage 1.119.O._10N.261.51.A9]	AUR89012.1	NCBI	1.00E-94
ORF34	Homeodomain-like protein	−	32903	33175	272	Homeodomain-like protein	AUR86879.1	NCBI	2.00E-28
ORF35	Nucleoside Triphosphate Pyrophosphohydrolase	−	33254	33796	543	[Superfamily] cl16941 (PSSMID 354290) Nucleoside Triphosphate Pyrophosphohydrolase (EC 3.6.1.8) MazG-like domain superfamily	cd11542	NCBI Conserved Domain Search	8.02E-27
ORF36	Adenine methylase	−	33923	34483	560	Adenine methylase [Aeromonas phage 4_D05]	QDJ96121.1	NCBI	3.00E-90
ORF37	DNA helicase	−	35354	36724	1370	Replicative DNA helicase [Vibrio phage jenny 12G5]	AGN51428.1	NCBI	0
ORF38	Hypothetical protein	−	36721	37425	705	Hypothetical protein ValSw33_44 [Vibrio phage ValSw3-3]	AVR75868.1	NCBI	1.00E-68
ORF39	Hypothetical protein	−	37488	37733	246	Hypothetical protein S349_62 [Shewanella sp. phage 3/49]	YP_009103948.1	NCBI	1.00E-10
ORF40	Hypothetical protein	−	37871	38242	372	Hypothetical protein VPR_009 [Vibrio phage Vp_R1]	AUG88373.1	NCBI	5.00E-54
ORF41	Hypothetical protein	−	38629	38919	291	Hypothetical protein NVP1113A_38 [Vibrio phage 1.113.A._10N.286.51.E7]	AUR88439.1	NCBI	5.00E-31
ORF42	ATPase	+	39897	40544	647	ATPase [Aeromonas phage 2_D05]	QDB73849.1	NCBI	2.00E-99
ORF43	Hypothetical protein	+	40525	41121	597	Hypothetical protein	166167	ACLAME	2.00E-26
ORF44	Fibrinogen binding protein	+	41169	42131	962	Fibrinogen binding protein [Vibrio phage 1.013.O._10N.286.54.F9]	AUR81803.1	NCBI	3.00E-137
ORF45	N-acetylmuramoyl-L-alanine amidase	+	42558	43019	461	N-acetylmuramoyl-L-alanine amidase [Vibrio phage 1.232.O._10N.261.51.E11]	AUR96787.1	NCBI	3.00E-51
ORF46	TMhelix containing protein	+	43209	43460	251	TMhelix containing protein [Vibrio phage 1.134.O._10N.222.52.B8]	AUR89889.1	NCBI	3.00E-23
ORF47	Transcriptional regulator	+	43798	44172	375	Transcriptional regulator	184491	ACLAME	5.00E-06
ORF48	Hypothetical protein	+	44476	45090	615	Hypothetical protein ValSw33_24 [Vibrio phage ValSw3-3]	AVR75848.1	NCBI	2.00E-28

ORFs with the predicted functions were determined by their significant hit (E-value < 10^−4^) against genome databases.

As shown in Table 2, we found that the majority of predicted ORFs were virion structural proteins including head morphogenesis protein, tail tape measurement protein, and tail tubular protein as well as ATPase and both small and large subunits of the terminase enzyme, which is involved in DNA encapsidation. A phylogenetic tree of large subunits of terminase of phage Seahorse revealed the close relationship to the temperate vibriophage MAR10 (Fig. S2). Phage Seahorse encoded a set of crucial enzymes that are involved in DNA replication and transcription (e.g. DNA helicase, ribonuclease, and transcriptional regulator), and DNA metabolism and modification (e.g. nucleoside triphosphate pyrophosphohydrolase and adenine methylase). Some ORFs were predicted as a transposase which is involved in phage DNA integration into the host genome while others were categorized as High frequency lysogenization C and Rha family proteins, which also serve a role in the regulation of lysogenic life cycle of phages, all of which suggest that phage Seahorse is indeed a temperate phage^36–38^. This annotation was further validated by a lysogeny experiment and a host cell lysis profile, both confirming that phage Seahorse has an ability to lysogenize the host (Fig. S3). Moreover, we also identify N-acetylmuramoyl-L-alanine amidase, an enzyme that degrades the peptidoglycan layer in bacterial cell walls^39^.

### Phage Seahorse infection triggers the condensation of host nucleoid

**Figure 3:**
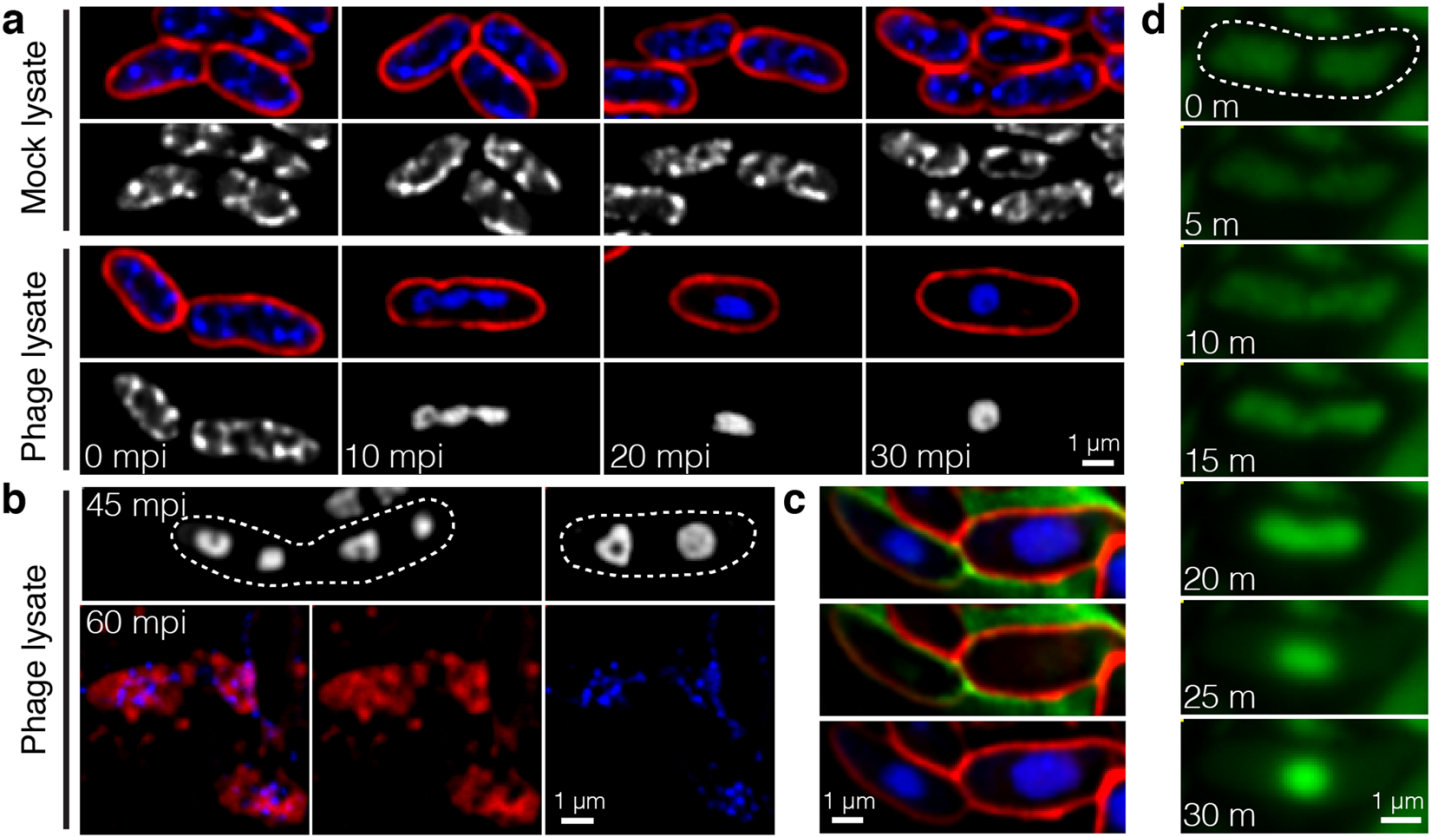
Single cell-leveled assay revealing the formation of blob and toroid of host DNA inside the phage-infected cells. Bacterial cells were grown in liquid culture to log phase and infected by phage Seahorse at MOI 5. At desired time points, the bacterial cells were harvested and fixed. For live cell imaging, the cells were inoculated on an agar pad after the phage infection. Prior to fluorescence microscopy, cell membrane (red) and nucleoid (blue/grey) were stained by FM4–64 and DAPI, respectively. (**a**) Fluorescence images of fixed bacterial cells in the presence of mock lysate (upper panel) and phage lysate (lower panel) at various time points. (**b**) Still images of phage-infected cells during late infection; 45 mpi (upper panel) and 60 mpi (lower panel). (**c**) Live cell images of phage-infected cells. SYTOX-green as impermeable DNA staining dye was used as an indicator of live cells. (**d**) Time-lapse imaging of phage-infected cells over the course of 30 minutes. Nucleoid (green) as stained by SYTO 16 condensed and became blob shaped as early as 25 minutes. Dashed lines indicate cell borders. Scale bars equal to 1 micron.

To investigate how phage Seahorse hijacks and kills the host VP_APHND_, a single cell-leveled assay was used to visualize the bacterial cells upon the phage infection. We first focused on a 30-minute post infection (mpi) window because the one-step growth curve suggests that the phage replicates inside the host for only approximately 30 minutes before cell lysis (Fig. 1g). Fluorescence microscopy of Seahorse infected VP_APHND_ revealed a nonuniformly distributed nucleoid at time zero (0 mpi, lower panel; Fig. 3a), identical to the uninfected VP_APHND_ control (0 mpi, upper panel; Fig. 3a). Over intervals of 10 mpi, this distributed nucleoid became more condensed as infection progressed until 30 mpi in which it appeared as a single sphere (30 mpi, lower panel; Fig. 3a). During late infection (after 30 mpi), some unlysed infected cells contained multiple nucleoids exhibiting an archetypal toroid shape (upper panel; Fig. 3b). Similar to a previous study in *Pseudomonas chlororaphis* phage 201Phi2-1, the bacterial host cells lysed at a late time point resulting in the release of phage particles appearing as puncta (blue) surrounding cell debris (red) suggesting the complete lytic cycle of the phage (lower panel; Fig. 3b)^40^. We observed no evidence of condensed DNA (“blob”) or toroid formation in the control cells (Figs. 3a and S4; n = 1,782).

To rule out the possibility that the observed nucleoid condensation is caused by host cell membrane leakage, previously seen in pore-forming molecules such as nisin and calcimycin^31^, we investigated the membrane integrity of infected cells by testing cell permeability to the DNA staining dye, SYTOX-green. Our result showed that the DNA blob in the infected cells was not stained by SYTOX-green, indicating that the cell membrane remained intact throughout infection, further supporting that phage Seahorse is the cause of the blob DNA formation, not the cell membrane leakage (Fig. 3c).

To confirm that the condensed nucleoid seen in the phage-infected cell is host bacterial DNA, we performed time-lapse fluorescence microscopy over a 30-minute infection period using live cell permeant SYTO 16 DNA dye. At the beginning of infection, the host nucleoid (green) appeared diffuse, similar to the uninfected cell control (Fig. 3d, Movie S2). Over the first 10 minutes, the nucleoid decondensed, seen by the diffusion and reduction of signal within the cell. Beginning at 15 mpi, the DNA nucleoid condensed and eventually appeared in a sphere at the midcell by 30 mpi, identical to the structure seen the infected cell shown in Fig. 3a. This time-lapse observation mirrors the morphological change in the DNA of fixed infected cells conducted over the same time course as shown in Fig. 3a. Altogether, these results suggest that phage Seahorse possibly interferes with the host cellular machineries in which it triggers the morphology change of host DNA.

### Inhibition of protein translation resulted in blob and toroid structure formation of the bacterial chromosome

**Figure 4:**
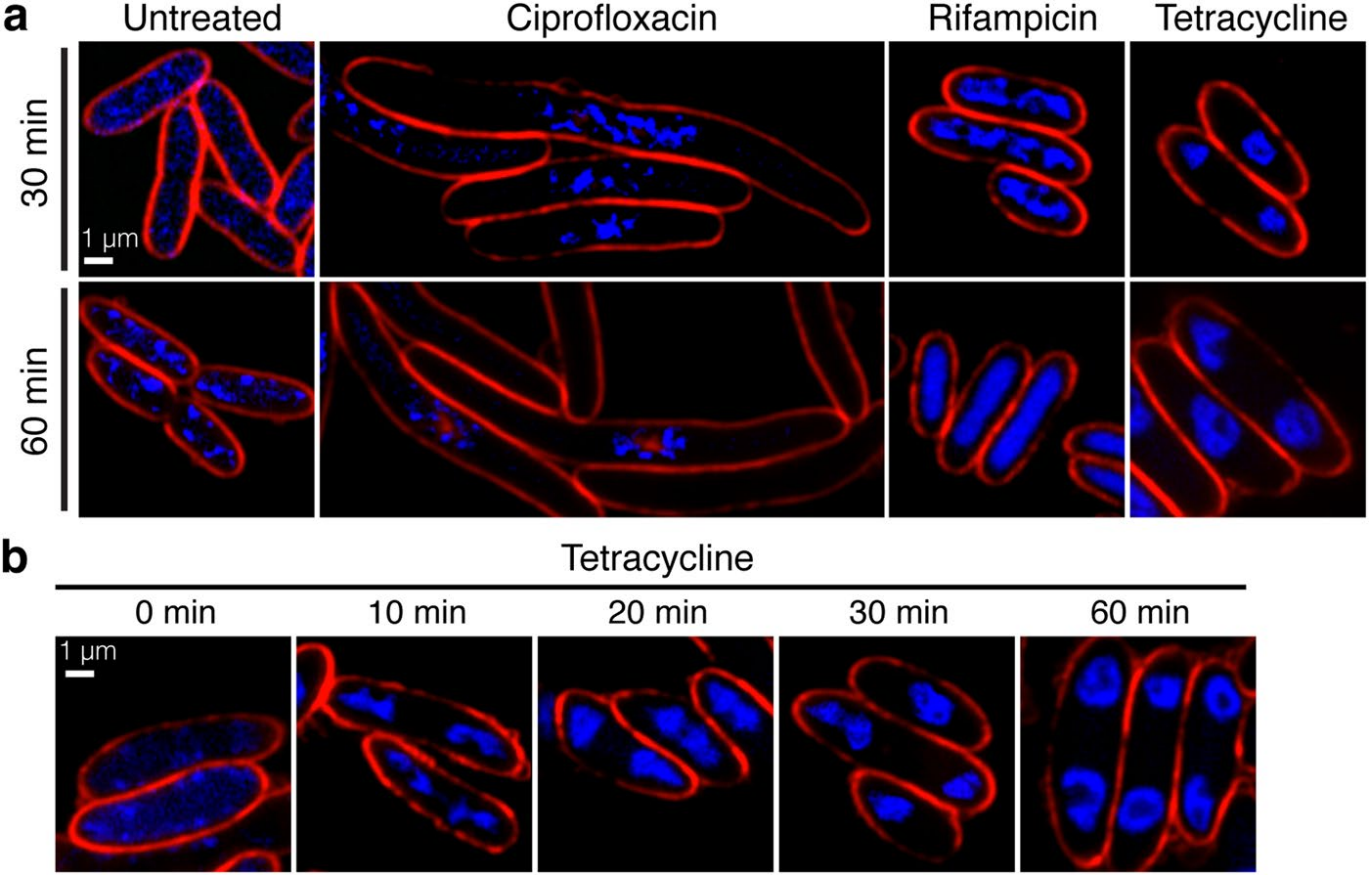
Fluorescence microscopy showed unique morphology of bacterial cells during the antibiotic treatment targeting different cellular pathways. Bacterial cells during the log phase were treated with antibiotics (ciprofloxacin, rifampicin, and tetracycline) at 5X MIC for indicated period of time. At desired time points, the cells were fixed and stained with FM4–64 (red) and DAPI (blue) prior to fluorescence microscopy. (**a**) Fluorescence images of fixed bacterial cells after the treatment with different antibiotics at 30 minutes (upper panel) and 60 minutes (lower panel). (**b**) Time-course still images of tetracycline-treated bacterial cells. Scale bars equal to 1 micron.

As previously reported by Nonejuie *et al*.^31^, fluorescence microscopy-based method, bacterial cytological profiling (BCP), can be used to identify biosynthetic pathways of bacteria that are inhibited based on characteristic cell morphological changes. We therefore applied the principle of BCP to investigate which metabolic pathway of VP_AHPND_ is targeted during the phage Seahorse infection by fluorescence microscopy. Since phages are believed to hijack host DNA replication, RNA transcription, and protein translation pathways during the lytic cycle^41^, we focused on antibiotics that also inhibit these major pathways. Ciprofloxacin, rifampicin, and tetracycline were used as inhibitors to replication, transcription, and translation respectively. Fluorescence microscopy results of VP_AHPND_ treated with antibiotics showed that each antibiotic treatment led to a unique morphological change in VP_AHPND_, similar to what previously observed in gram-negative *Escherichia coli* and *Acinetobactor baumannii* treatments^31,42^. Upon ciprofloxacin treatment, inhibition of DNA replication resulted in cell elongation and DNA pooling at the midcell while treatment with rifampicin resulted in DNA decondensation (Fig. 4a). Tetracycline-treated cells exhibited the signature condensed and toroidal-shaped DNA at 30 minutes but more prominently at 60 minutes post treatment with an intact cell membrane (Figs. 4a and S5). This morphology upon tetracycline treatment is notably similar to the condensed DNA morphology of the bacterial cells infected by phage Seahorse (lower panel; Fig. 3a).

The production of “condensed” DNA upon infection instead of perfect “toroidal” shaped DNA urged us to ask if the condensed DNA morphology is truly the result of protein translation inhibition (lower panel, 10–30 mpi; Fig. 3a). The archetypal DNA shape of protein translation inhibition in many studies is a “toroid”^31,42,43^, but DNA shape alteration during very early translation inhibition by an antibiotic (less than one hour) has never been reported. Thus, time-course analysis of toroidal DNA formation in VP_AHPND_ during tetracycline treatment was performed. This resulted in a more “condensed” DNA morphology that changed over time with the eventual appearance of the signature “toroid” DNA at 30 minutes post treatment and it became increasingly prominent at 60 minutes (Fig. 4b). Due to the fact that the phage Seahorse had a short latent period and completed its lytic cycle by 30 mpi, it is then reasonable to assume that the toroid DNA morphology was merely undetectable in infected cells because the host cell lysed before toroids were formed. Simply put, if it was able to progress beyond 30 mpi, the nucleoid would have resembled a toroid shape seen in Fig. 4b (45 mpi; Fig. 3b). Altogether, these results suggest that DNA condensation morphology observed in phage Seahorse-infected VP_AHPND_ is likely due to the protein translation inhibition caused by the phage hijacking mechanism.

## Discussion

A new strain of *V. parahaemolyticus*, called VP_APHND_, emerged in 2009 as a devastating pathogen to shrimp, causing acute hepatopancreatic necrosis disease (AHPND) or early mortality syndrome (EMS). The infection resulted in mass production losses in southern China and within several years, the incidence of VP_APHND_ infection had expanded to other neighboring countries such as Vietnam, Malaysia and Thailand^11^. The disease contributes to 100% mortality of shrimp within one week and the infected animals present an atrophied and discolored hepatopancreas due to the *Photorhabdus* insect-related (Pir) binary toxins (PirA^*vp*^/PirB^*vp*^), which are encoded on the virulent plasmid of the pathogen. The toxins degenerate the tubule epithelial cells of the hepatopancreas leaving the diseased animals susceptible to additional bacterial infections^44,45^.

Due to the emergence of VP_AHPND_ and its multidrug resistant isolates, effective tools to control and combat these pathogens are urgently needed. Most recently, Angulo *et al*. revealed a number of studies and research reports on using phages as a biocontrol for the wide spread of AHPND^26^. Until now, even though many phages have been reported to target *V. parahaemolyticus*, only phage pVp-1 has been found to effectively kill VP_AHPND_. Phage pVp-1 is highly potent and lyses more than 90% out of the VP_AHPND_ strains that were isolated from Vietnam, Mexico, Costa Rica, Honduras, and Central America Countries^29^. However, the bacterial strain isolated from Thailand is not targeted by the pVp-1 phage. In this study, we successfully isolated a novel phage (named “Seahorse”) that specifically infects the VP_AHPND_ strain isolated in Thailand. This study also elucidates that the phage is able to hijack protein translation machinery of the host bacteria. In comparison to other phages that infect vibrios (Table S2)^46–52^, phage Seahorse has relatively big burst size with a shorter latency period than phages VP-1, VP-2, VP-3, VpKK5, and VhKM4 that replicate in *V. parahaemolyticus*^27,29,53–56^. It is also tolerant to the wide range of pHs and temperatures compared to other vibriophages suggesting unique survivability in harsh natural environments. These infectious characteristics not only add phage Seahorse to the library of phage targeting VP_AHPND_ but also make it a potential candidate for a biocontrol agent.

Bacteriophage requires a bacterial host cell to replicate, manipulating the native biosynthesis machinery of the host to assemble its own progeny. During its reproduction process, various phage-encoding proteins are produced to redirect or inhibit the host metabolism at the molecular level to benefit its fitness. These phage-derived proteins not only target the major metabolic pathways but are also capable of lysing the host cell membrane. Due to nature of these effects, these proteins can be considered antimicrobial agents similar to that of antibiotics with possible therapeutic applications^39^. Phage Seahorse was classified in the family *Siphoviridae* due to characteristics of the flexible, noncontractile tail with a non-enveloped icosahedral capsid. In addition, at high resolution near a native-state by Cryo-ET, unique spikes and fibers (a shaft with a sphere at the tip) on the phage capsid were observed. This is not the first time that this structure has been reported^32–35^. These are most likely structurally diverse glycoproteins encoded by the phage and thought to promote tethering of the viral capsid to receptors present on the host cell surface. For instance, in the case of human adenoviruses, the fibers have a long shaft with a knob at the distal end of the capsid whereas, in phage Phi29 and some bovine adenoviruses, they consist of only a protruding stem without a terminal sphere^32,34,35,57,58^. Moreover, phage Sf6 that infects *Shigella flexneri* has an identical shaft-knob structure at the tail terminus called the “tail needle knob”^59^. Due to the narrow host spectrum of phage Seahorse, the capsid spikes and fibers located on the surface of the phage capsid serve a potential role in host specificity and recognition.

Unfortunately, in a therapeutic context, phage Seahorse is not appropriate for application due to the ability to enter lysogenic cycle which renders the phage unsafe for treatment. Phage Seahorse appeared phylogenetically related to the temperate phage MAR10 that belongs to the genus *Ssp2virus* and targets *V. parahaemolyticus* (Fig. S2)^60,61^. This reassures our conclusion that phage Seahorse is temperate and also suggests the possible viral family it belongs to. Moreover, due to the lack of bioinformatics in phage related databases, the majority of the ORFs annotated in the phage Seahorse genome were predicted as unknown. As they might produce unwanted products or other unknown virulence factors, the therapeutic application is not warranted unless the fundamental knowledge in phage biology is better established or the phage is engineered to strictly enter only the lytic cycle^62^.

However, despite the therapeutic shortcomings of phage Seahorse itself, phage genomes generally are considered an untapped resource for antimicrobials due to the metabolic hijacking ability and lytic capacity of the proteins they encode^63,64^. For example, from our genome analysis, albeit its relatively small genome, we were able to identify a lysis-related enzyme, N-acetylmuramoyl-L-alanine amidase, from ORF45. This lysis enzyme is involved in the cleavage of a very common bond present in most bacterial cell membranes, thus serving a crucial role in cell lysis^39^. In fact, it has been reported that the application of recombinant phage lysis enzymes from vibriophages can target a wider spectrum of bacterial hosts as compared to the parental phages^65,66^. Therefore, there is a strong possibility of finding other antibacterial protein candidate from this small phage in the future. Determining whether or not other hypothetical proteins found in the phage Seahorse genome and other newly discovered phage exhibit antibacterial activity needs further investigation.

With the lysis-related enzyme in mind, we set out to investigate whether the phage Seahorse genome encoded other proteins that targeted major host metabolic pathways during its lytic cycle. Bacterial morphological changes have been used as indicators for various physiological states of bacteria elicited by genetic alteration or stress response^31,67,68^. BCP exploits these cell morphological change patterns under different stresses to identify the specific mechanism being targeted by an antibiotic that causes bacterial growth inhibition. This study, for the first time, applied the principle of BCP technique to identify the underlying effected pathway and found that phage Seahorse likely inhibits protein translation of VP_AHPND_ at the early stage of infection. This finding is concomitant with other reported hijacking mechanisms that use phage-host protein-protein interactions to inhibit host machineries (i.e. replication, transcription and translation) with the effect of disarming host defenses and producing its own proteins for reproduction^69^. Thus, the hijacking model of host protein machinery in favor of phage protein production right after infection is plausible. For example, *Pseudomonas aeruginosa* phage PaP3 strongly suppresses host protein synthesis through the reduction of ribosome to preserve energy^70^. Our finding does not directly indicate that native host replication and transcription are not also inhibited during the infection. To date, BCP has never been applied to study replication, transcription and translation inhibition simultaneously or chronologically. Thus, it is possible that host replication and transcription machineries were inhibited but only the profound protein translation inhibition phenotype was detected. Whether or not a temporal hijacking mechanism is presented during different stages of infection requires further investigation.

Our study revealed the formation of host toroidal nucleoids that are likely the result of encoded phage proteins that hijack host translation machinery before cell lysis, as we called it: “Mechanism of pre-killing (MOK)”. This study suggests that the phage Seahorse genome contained at least one product that was involved in the inhibition towards a protein biosynthesis pathway. Further investigation into which of the phage-derived proteins target important pathways of the bacterial host will be needed in order to identify these antimicrobial proteins for development as therapeutics against pathogens. Localization profiling of phage proteins inside the host, as we previously reported^71,72^, could also be utilized to study Seahorse-infected cells to gain a better understanding of how individual phage-encoded proteins temporally and spatially function within the host. These investigations could help overcome Seahorse’s therapeutic shortcomings while identifying novel antimicrobial agents at the molecular level.

## Material and Methods

### Phage isolation, purification, and preparation

Overnight cultures of VP_AHPND_ were prepared by inoculating the bacteria, previously grown on Tryptic Soy Agar supplemented with 1.5% Sodium chloride (TSA-1.5%NaCl), into 5 ml of Tryptic Soy Broth supplemented with 1.5% Sodium chloride (TSB-1.5%NaCl) and allowed to incubate at 30 °C, 200 rpm for at least 16 hours. One milliliter of seawater was added to 25 ml of TSB-1.5%NaCl, 250 μl of 100 mM CaCl_2_, and 2.5 ml of VP_AHPND_ overnight culture. Phage was enriched by growing at 30 °C, 200 rpm for 48 hours. Phage was next harvested by centrifugation at 9,000 rpm for 10 minutes followed by collecting and filtering the supernatant using a 0.45 μm filter. Next, phage purification was performed using double-layer agar method. Briefly, 10-fold serial dilutions of phage were prepared using SM buffer. Ten microliters of each diluent were added to 100μl of overnight culture of VP_AHPND_, mixed and allowed to stand for 10 minutes. To this, 5 ml of melted 0.35% top agar of TSA-1.5%NaCl was added, mixed and poured onto a TSA-1.5%NaCl plate. The plates were incubated overnight at 30 °C. Putative translucent plaques were identified and picked and resuspended in 100 μl of SM buffer. This purification step was repeated 3 times. A high-titer phage lysate was prepared described by Chaikeeratisak *et al*.^72^. Briefly, 5 ml SM buffer was added to a near-confluent plate and incubated at 30 °C for at least 5 hours. The solution was aspirated into a tube and centrifuge at 9,000 rpm for 5 minutes. Finally, the supernatant was filtered using a 0.45 μm filter and stored at 4 °C.

This work has been reviewed and approved by Chulalongkorn University-Institutional Biosafety Committee (CU-IBC) in accordance with the levels of risk in pathogens and animal toxins listed in the Risk Group of Pathogen and Animal Toxin (2017) published by Department of Medical Sciences (Ministry of Public Health), the Pathogen and Animal Toxin Act (2015) and Biosafety Guidelines for Modern Biotechnology BIOTEC (2016) with approval number: SC CU-IBC-006/2018.

### Transmission electron microscopy and cryo-electron tomography

3 μl of phage titer was deposited on QUANTIFOIL 200 mesh holey carbon R 2/1 gold grids, glow-discharged using PELCO easiGlow (Ted Pella). The grids were blotted using Whatman No. one filter paper and plunge-frozen into a liquid ethane/propane mixture cooled by liquid nitrogen using a custom-built device (Max Planck Institute for Biochemistry, Munich). Tilt series were collected on grids clipped onto autogrids (Thermo Scientific) in a 300 keV Titan Krios (Thermo Scientific) fitted with a K2 Summit 4k x 4k pixel direct electron detector and a GIF Quantum post-column energy filter (Gatan) using a nominal magnification of 42 kx or a pixel size of 3.4 Å and −5 μm defocus. Tilt series were acquired using SerialEM in low dose mode, typically from −40o to +40o every 2–3 degrees with a total dose of 50–70 e/Å2. The tilt series were aligned and dose-weighted according to the cumulative dose using MotionCor2 and reconstructed in IMOD software using weighted back-projection.

### Conventional phage study

To evaluate phage adsorption, VP_AHPND_ culture (OD_600_ ~ 0.4) was infected with phage particles at MOI 0.01 (The OD_600_ of 1 = 1 × 10^9^ CFU/ml^73^) and incubated at 30 °C. At each time point of 0, 1, 2, 5, 7.5, 10, 15, 20, 25, and 30 minutes, 100 μl of the samples were collected and diluted 10-fold in SM buffer. After centrifugation at 15,000 × g for 2 minutes at 4 °C, the supernatant was harvested and the number of free phages was determined by double-layer agar method. For the one-step growth curve analysis, VP_AHPND_ was infected at MOI 0.01 at 30 °C for 15 minutes, then the cell suspension was centrifuged at 12,000 × g for 5 minutes. The pellet was resuspended in 10 ml of TSB-1.5% NaCl. The mixture was then incubated with vigorous shaking at 200 rpm, 30 °C for 2 hours. Throughout the period of shaking, the samples of the untreated group and the chloroform-treated group were taken every 10 minutes to evaluate total virions by double-layer agar method.

For pH stability, 100 μl of phage lysate was mixed with 900 μl of SM buffer in a pH range 2 to 10 and incubated at 30 °C for 1 hour. For temperature stability, 50 μl of phage lysate was incubated for 1 hour at different temperatures; 4, 20, 25, 30, 37, 40, 50, 60, and 70 °C. For both tests, phage infectivity was determined by performing a spot test. These experiments were performed in triplicate.

To determine host spectrum of the phage, a spot test was performed to test the infectivity of the isolated phage against different 26 bacterial strains that we obtained and were kindly offered from different sources as indicated in Table 1. Briefly, overnight cultures were prepared as described above. 500 μl of each culture was mixed with 5 ml of 0.35% molten top agar (TSA-1.5% NaCl) and immediately poured on an agar plate (TSA-1.5% NaCl). After the cell lawn was solidified, 5 μl of each diluent of 10-fold serially diluted phage was spotted on the surface of the top agar. The plates were allowed to dry and next incubated overnight at 30 °C. The clearing zones were then evaluated for the infection ability of the phage toward the bacterial host.

### Phylogenetic tree construction

DNA sequences of the terminase large subunit of various phages were obtained through GenBank. These sequences included accession number and phage names as follows: Vibriophage KVP40: NC_005083, Vibriophage CP-T1: NC_019457.1, Vibriophage pVp-1: NC_019529.1, Vibriophage vB_VpaS_MAR10: NC_019713.1, Vibriophage VH7D: NC_023568.1, *Enterobacteria* phage 9 g: NC_024146.1, *Salmonella* phage Stitch: NC_027297.1, Vibriophage phi 3: NC_028895.1, *Enterobacteria* phage JenK1: NC_029021.1, *Vibrio vulnificus* phage SSP002: NC_041910, *Enterobacteria* phage EPS7: NC_010583.1 and phage Seahorse. The sequences were aligned using ClustalW and the phylogenetic tree was constructed using Molecular Evolutionary Genetics Analysis (MEGA) version 10.0 as described by Kumar *et al*.^74^. Using the Maximum Likelihood method, a bootstrap consensus phylogenetic tree from 100 bootstrap replications for tree construction was selected. The selected numbers of bootstrap were shown on the selected branches.

### Lysogeny experiment

To isolate phage-resistant strains, bacterial colonies that appeared in a double layer-agar plate at high titer of phage were picked and further isolated. The isolated strains and original VP_AHPND_ were tested for phage resistance by cross streaking each isolate with a drop of high titer phage lysate atop the bacterial stripes. The plate was incubated overnight at 30 °C and the result was recorded. To test whether the phage-resistant isolates were lysogen, a bacterial cell lawn of VP_AHPND_ strain was prepared as described above. Single colony of each phage-resistant isolate was picked by a sterile toothpick and stabbed into the top agar. The plate was then incubated at 30 °C overnight and the presence of a clear zone surrounding the stab isolate was recorded the day after.

### Bacterial cell lysis profile assay

VP_AHPND_ culture at mid-log phase (OD_600_ ~ 0.4) was inoculated with phage lysate at MOI 0 as a control and MOI 0.01 and MOI 5 as experimental groups. The cultures were then incubated shaking at 200 RPM at 30 °C. OD_600_ of all cultures were monitored every 30 minutes until 10 hours of incubation. The experiment was carried out in triplicate.

### Phage genome DNA extraction

The phage lysate was first dialyzed in sterile distilled water. Next, phage was precipitated by adding 2.5 ml of phage precipitant solution (30% w/v PEG-8,000, 3.3 M NaCl and sterile distilled water) to 10 ml of phage lysate (~10^9^ pfu/ml) and stored overnight at 4 °C. The solution was then centrifuged at 10,000 rpm for 30 minutes followed by resuspending the pellet in 500 μl of 1xDNase I buffer. To degrade bacterial genomic DNA and RNA, 5U DNaseI and 25 μg RNaseA were added and incubated at 37 °C for 2 hours. Next, 25 mM EDTA was added to inhibit nuclease activity, followed by 0.5% SDS and 25 μg proteinase K, and incubated at 60 °C for 2 hours. Phenol-chloroform extraction was then performed to extract phage genomic DNA.

### Phage genome sequencing and analysis

Phage genomic DNA was sequenced by Illumina MiSeq platform. All raw reads were qualified and the low qualities were eliminated. The adaptors in the filtered reads were then trimmed and assembled into contigs. To remove potential host DNA contamination, reads were mapped to the host strain sequence of *V. parahaemolyticus* strain ATCC17802 (GenBank accessions CP014046, CP014047) using the Geneious mapper in Geneious Prime 2019 (https://www.geneious.com). The unmapped reads were used for assembly in Geneious Prime 2019 with the Geneious assembler using high sensitivity and default parameters. A list of ORFs was generated from this contig using the ORF finder in Geneious; filtering out any ORFs less than 200 base pairs. The protein sequences of each ORFs were predicted by EMBOSS Transeq and they were annotated manually by BLASTp and PSI-BLAST (cut-off e-value < 10^−4^) against various databases: NCBI’s non-redundant (nr) protein sequences, InterPro 75.0, NCBI conserved domain and ACLAME. In addition, to confirm the predicted function, RAST sever, Prodigal and PHASTER were used as well. To determined antimicrobial resistance coding genes and putative toxins, RESFINDER and VirulenceFinder were used, respectively. Aragon and tRNAScanSE were used to identify tRNAs. The map of genome was drawn by Artemis and DNA plotter.

### Single cell-infection assay

VP_AHPND_ culture (OD_600_ ~ 0.4) was infected with phage at MOI 5 and the infected cells were incubated at 30 °C. At each time point; 0, 10, 20 and 30 minutes, the samples were harvested by centrifugation at 9,000 rpm for 2 minutes and the supernatant discarded. As described by Chaikeeratisak *et al*.^72^, phage-infected cells were fixed at a final concentration of 4% paraformaldehyde and incubated at room temperature for 15 minutes. The fixed cells were centrifuged and the pellets were washed with 500 μl of 1x PBS three times. The cells were resuspended in 1x PBS before loading 3 μl onto an agarose pad (1.2% agarose in 20% TSB-1.5% NaCl) that contained fluorescent dyes (2 μg/ml FM 4–64 and 2 μg/ml DAPI). The samples were visualized under DeltaVision Ultra High-Resolution Microscope. For live cells, the cells were harvested at desired time points, and inoculated on an agarose pad as described above. The nucleoid was stained with either 0.5 μM SYTOX-green or 0.5 μM SYTO 16, prior to fluorescence microscopy.

### Minimal inhibitory concentration

Minimal inhibitory concentrations (MIC) were determined for the following antibiotics: Ciprofloxacin, Rifampicin, and Tetracycline, which were all used in the fluorescence microscopy experiment shown in Table S1. The antibiotics were respectively serially diluted in a 96 well plate using a microdilution method^42^. Overnight cultures of VP_AHPND_ were diluted 100-fold in TSB-1.5% NaCl and allowed to grow on a roller at 30 °C until exponential growth (OD_600_ of 0.2) was observed. The culture was further diluted 100-fold in TSB-1.5% NaCl into wells of the same 96-well plate that contained different concentrations of the respective antibiotic. The culture was further incubated at 30 °C for 24 hours. MICs for each antibiotic were determined as the lowest concentration dilution of that antibiotic capable of inhibiting growth of the bacteria.

### Fluorescence microscopy

Overnight cultures of VP_AHPND_ were diluted 100-fold in TSB-1.5% NaCl and incubated at 30 °C on a roller until exponential phase of growth was obtained. Antibiotics were added to the culture at concentrations of 5 times the MIC. For live cell imaging, cultures were incubated at 30 °C on a roller for 60 minutes followed by staining with fluorescent dyes; FM 4–64 (2 μg/ml), DAPI (2 μg/ml) and SYTOX-green (0.5 μM). Cultures were then harvested by centrifugation at 6,000 g for 30 seconds and resuspended in 30 μl of supernatant. 3 μl of sample was loaded onto agarose pad (1.2% agarose containing 20% TSB-1.5% NaCl) on concave glass slides and fluorescence microscopy was performed, following consistent imaging parameters throughout all of the experiments. For fixed cell imaging, cultures were incubated on a roller at 30 °C for 30 and 60 minutes for treatment with ciprofloxacin and rifampicin, while 10, 20, 30 and 60 minutes for treatment with tetracycline. After the completion of each treatment, cultures were fixed as described above. Cultures were then harvested by centrifugation at 9,000 rpm for 2 minutes followed by washing the pellet with 1x PBS for 3 times. After centrifugation, the pellet was resuspended in 30 μl of 1x PBS and added to an agarose pad as described above. Fluorescence microscopy was performed using consistent imaging parameters for all experiments.

## Supplementary information

Supplementary information.

Supplemental Movie 1.

Supplemental Movie 2.

## Data Availability

All data generated or analyzed in this study are included in this article and its supplementary information files. Nucleotide sequence of the phage Seahorse genome was deposited in GenBank database with the accession number MN512538.
