# Supplementary material for: A novel vibriophage exhibits inhibitory activity against host protein synthesis machinery
Source: Sci Rep. 2020 Feb 11;10:2347. doi: 10.1038/s41598-020-59396-3 (PMC7012835; doi:10.1038/s41598-020-59396-3)
Supplement: Supplementary file 1 — Supplementary information. [file 41598_2020_59396_MOESM1_ESM.pdf]

Supplementary Information

A novel vibriophage exhibits inhibitory activity against host protein synthesis machinery

Khrongkhwan Thammatinna[1*], MacKennon E. Egan[2*], Htut Htut Htoo[3], Kanika Khanna[2], Joseph Sugie[2], Jason F. Nideffer[2], Elizabeth Villa[2], Anchalee Tassanakajon[1], Joe Pogliano[2], Poochit Nonejuie[3], Vorrapon Chaikeeratisak[1**]

[1] Center of Excellence for Molecular Biology and Genomics of Shrimp, Department of Biochemistry, Faculty of Science, Chulalongkorn University, Bangkok, 10330, Thailand.

[2] Division of Biological Sciences, University of California, San Diego, La Jolla, California, USA.

[3] Institute of Molecular Biosciences, Mahidol University, Salaya, Nakhon Pathom, Thailand.

[*] These authors contributed equally.

[**] Correspondence: vorrapon.c@chula.ac.th.

Figure S1

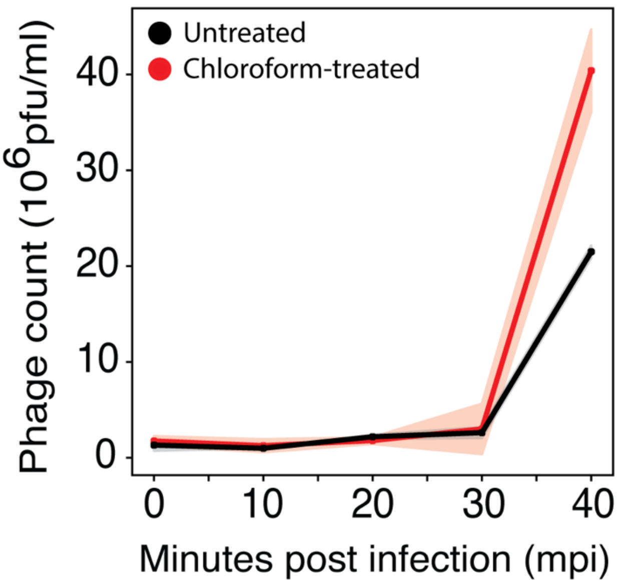

Supplemental Figure 1: One-step growth curve of phage Seahorse in comparison between untreated (black line) and chloroform-treated samples (red line). The experiment was performed in at least 3 independent biological replicates and the data are represented as the mean ± standard deviation.

Figure S2

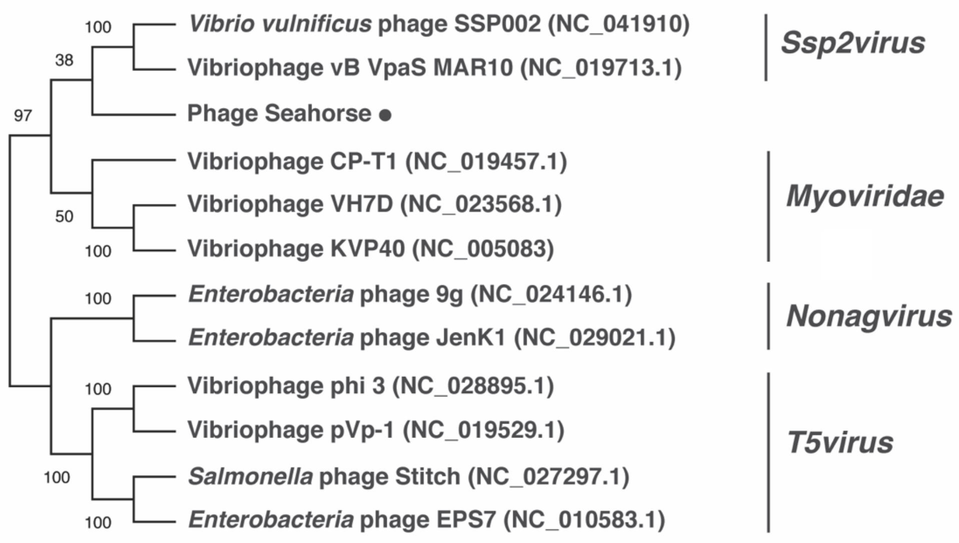

Supplemental Figure 2: Phylogenetic tree showing the relationship between terminase large subunits of different phages as indicated in the tree. Selected bootstrap values are shown at major branches. The viral classification: *Ssp2virus*, *Myoviridae*, *Nonagvirus*, and *T5virus*, is shown at each clustered group. A closed circle indicates the branch of terminase large subunit of phage Seahorse.

Figure S3

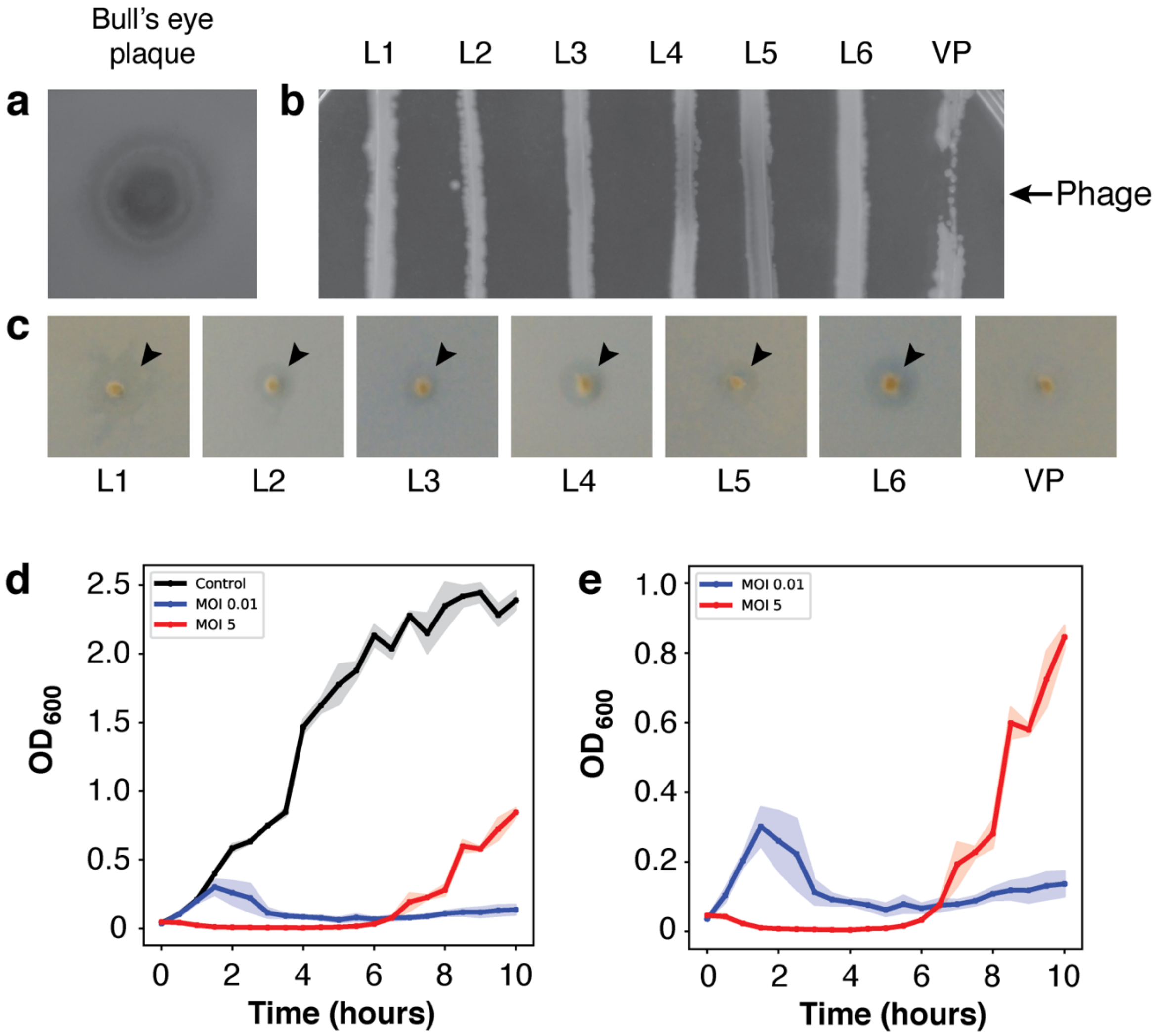

Supplemental Figure 3: Lysogeny experiment of phage Seahorse. (a) A spot test exhibiting a signature clear zone of temperate phages - "Bull's eye". (b) A cross-streak experiment of phage resistant isolates (L1 – L6) and the bacterial control (VP). Arrow indicates where high titer phage lysate was spotted atop the streaks. (c) Production of phage progeny from isolated lysogens. Arrowheads indicate clear zones as a result of host cell lysis. (d,e) The bacterial cell lysis profile of VP_{AHPND} in the presence of phage at MOI 0 (Control, black line), MOI 0.01 (blue line), and MOI 5 (red line).

Figure S4

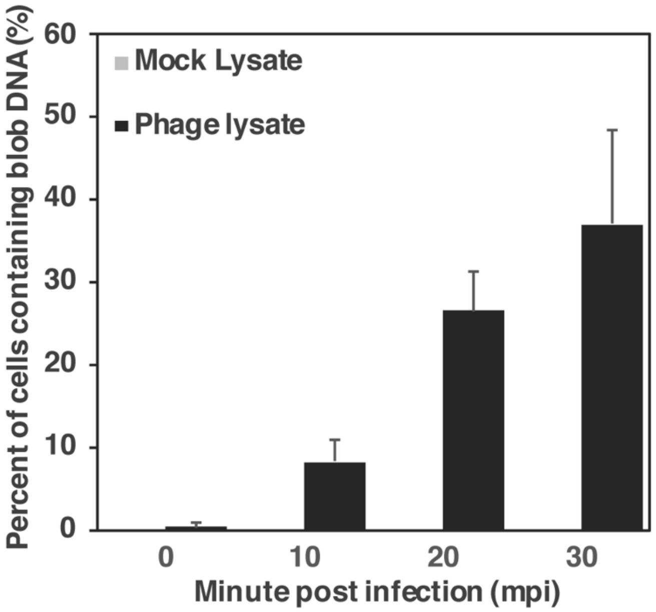

Supplemental Figure 4: A graph showing the increase of number of VP_{AHPND} cell containing blob-shaped DNA during the phage Seahorse infection.

Figure S5

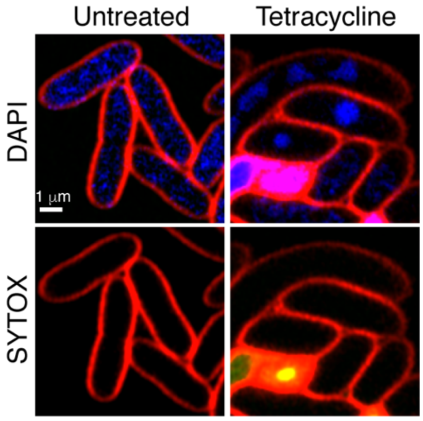

Supplemental Figure 5: Fluorescence images of live cells of VP_{AHPND} treated with tetracycline. Bacteria cells were treated with tetracycline for 60 minutes. SYTOX-green was used to stain DNA as an indicator for the cells with permeabilized membrane. Upper panels show FM4-64 (red) and DAPI (blue) staining while lower panels show FM4-64 (red) and SYTOX-green (green) stains. Scale bar equals to 1 micron.

Table S1: Antibiotics used in this study showing antibiotic class, MIC and drug target.

Antibiotic Class	Antibiotic Name	MIC (µg/ml)	Target
**DNA Synthesis Inhibitor**			
Fluoroquinolone	Ciprofloxacin	0.375	DNA gyrase A
**RNA Transcription Inhibitor**			
Rifamycin	Rifampicin	0.375	DNA-dependent RNA polymerase
**Protein Synthesis Inhibitors**			
Tetracycline	Tetracycline	0.5	30S ribosome (inhibit aminoacyl tRNA binding)

Table S2: A summary table of latent period, burst size, and tolerance of selected vibriophages

Phage name	Host	Stains	Latent Period (Minutes)	Burst Size (Particle/Cell)	Infectivity at pH	Infectivity at Temp (°C)	Reference
VP-1	*V. parahaemolyticus*	Non-specified strain	120	9	-	-	Mateus *et al.* (2014)[27]
VP-2	*V. parahaemolyticus*	Non-specified strain	90	15	-	-	Mateus *et al.* (2014)[27]
VP-3	*V. parahaemolyticus*	Non-specified strain	40	42	-	-	Mateus *et al.* (2014)[27]
VpKK5	*V. parahaemolyticus*	ATCC17802 and 15 isolates	36	180	4 to 9	40	Lal *et al.* (2016)[46]
VhKM4	*V. parahaemolyticus*	ATCC 17802	60	52	-	-	Lal *et al.* (2017)[47]
VVP1	*V. parahaemolyticus*	N1A and N7A	20-30	-	6 to 12	4 to 55	Stalin and Srinivasan (2016)[48]
pVp-1	*V. parahaemolyticus*	AHPND (22 strains)	15	47	5 to 11	20 to 50	Jun *et al.* (2016)[29]
ϕVP-1	*V. parahaemolyticus*	SV4	10	44	7 to 9	4 to 70	Matamp and Bhat (2019)[49]
vB_ValP_IME271	*V. alginolyticus*	1651	90	40	8	40	Li *et al.* (2019)[70]
Vp670	*V. alginolyticus*	E06333	30	84	-	-	Luo *et al.* (2018)[71]
Φa318	*V. alginolyticus*	ATCC 17749	15	72	-	lower than 50	Lin *et al.* (2012)[72]
pVa-21	*V. alginolyticus*	rm-8402	70	58	5 to 9	4 to 50	Kim et al. (2019)[73]
vB_VspP_pVa5	*V. splendidus*	VaAn	30	24	-	-	Katharios et al. (2017)[74]
H188	*V. kanaloae*	LMG 20539(T)	96	3	4 to 12	30 to 70	Li *et al.* (2016)[75]
BONAISHI	*V. coralliilyticus*	LMG20984	120-180	8 and 19	3 to 10	4 to 50	Jacquemot *et al.* (2018)[76]
**Seahorse**	***V. parahaemolyticus***	**AHPND (TM)**	**30**	**72**	**4 to 10**	**20 to 60**	**This study**

Supplemental Movie legends

Supplemental Movie 1: Movie showing different slices of a tomogram capturing the phage Seahorse depicted in Figure 1.
Supplemental Movie 2: Movie showing time-lapse imaging, related to Figure 3. Images of a phage Seahorse-infected cell were taken every 5 minutes for a total of 30 minutes.
